# Suppression of Mcl-1 via RNA interference sensitizes human hepatocellular carcinoma cells towards apoptosis induction

**DOI:** 10.1186/1471-2407-6-232

**Published:** 2006-10-02

**Authors:** Henning Schulze-Bergkamen, Binje Fleischer, Marcus Schuchmann, Achim Weber, Arndt Weinmann, Peter H Krammer, Peter R Galle

**Affiliations:** 1 First Department of Medicine, Johannes-Gutenberg-University Mainz, Langenbeckstrasse 1, 55101 Mainz, Germany; 2 Institute of Pathology, Johannes-Gutenberg-University Mainz, Langenbeckstrasse 1, 55101 Mainz, Germany; 3 German Cancer Research Center, Tumor Immunology Program, Heidelberg, Germany

## Abstract

**Background:**

Hepatocelluar carcinoma (HCC) is one of the most common cancers worldwide and a major cause of cancer-related mortality. HCC is highly resistant to currently available chemotherapeutic drugs. Defects in apoptosis signaling contribute to this resistance. Myeloid cell leukemia-1 (Mcl-1) is an anti-apoptotic member of the Bcl-2 protein family which interferes with mitochondrial activation. In a previous study we have shown that Mcl-1 is highly expressed in tissues of human HCC. In this study, we manipulated expression of the Mcl-1 protein in HCC cells by RNA interference and analyzed its impact on apoptosis sensitivity of HCC cells *in vitro*.

**Methods:**

RNA interference was performed by transfecting siRNA to specifically knock down Mcl-1 expression in HCC cells. Mcl-1 expression was measured by quantitative real-time PCR and Western blot. Induction of apoptosis and caspase activity after treatment with chemotherapeutic drugs and different targeted therapies were measured by flow cytometry and fluorometric analysis, respectively.

**Results:**

Here we demonstrate that Mcl-1 expressing HCC cell lines show low sensitivity towards treatment with a panel of chemotherapeutic drugs. However, treatment with the anthracycline derivative epirubicin resulted in comparatively high apoptosis rates in HCC cells. Inhibition of the kinase PI3K significantly increased apoptosis induction by chemotherapy. RNA interference efficiently downregulated Mcl-1 expression in HCC cells. Mcl-1 downregulation sensitized HCC cells to different chemotherapeutic agents. Sensitization was accompanied by profound activation of caspase-3 and -9. In addition, Mcl-1 downregulation also increased apoptosis rates after treatment with PI3K inhibitors and, to a lower extent, after treatment with mTOR, Raf I and VEGF/PDGF kinase inhibitors. TRAIL-induced apoptosis did not markedly respond to Mcl-1 knockdown. Additionally, knockdown of Mcl-1 efficiently enhanced apoptosis sensitivity towards combined treatment modalities: Mcl-1 knockdown significantly augmented apoptosis sensitivity of HCC cells towards chemotherapy combined with PI3K inhibition.

**Conclusion:**

Our data suggest that specific downregulation of Mcl-1 by RNA interference is a promising approach to sensitize HCC cells towards chemotherapy and molecularly targeted therapies.

## Background

The incidence of hepatocellular carcinoma (HCC) in Western countries has experienced a significant increase over recent years. Currently, HCC ranks among the five most important causes of cancer-related mortality worldwide [1]. In Western countries, HCC occurs mainly in patients with liver cirrhosis and has an annual incidence of about 2–4 cases per 100,000. In developing countries, the incidence is approximately 20/100,000. The increasing incidence of HCC is mainly due to the large number of HCV-seropositive patients. Most patients with HCC show advanced-stage tumor at the time of diagnosis, and therefore, curative surgical treatment can only be achieved in a minority of patients [2]. The therapeutical options for palliative treatment as well as in patients awaiting liver transplantation are rare [3]. Therefore, new treatment regimens for patients with advanced HCC are needed.

Defects in apoptosis signaling contribute to tumorigenesis and chemotherapy resistance of HCC cells. Stabilization of mitochondrial integrity is a key mechanism for both the survival of a malignant cell and for its resistance to chemotherapy [4,5]. A well established family of proteins that has a significant impact on mitochondrial integrity by influencing the permeability of the mitochondrial membrane is the Bcl-2 family. Bcl-2 family members can be roughly subdivided into anti- and pro-apoptotic proteins. Myeloid cell leukemia-1 (Mcl-1) is an anti-apoptotic member of the Bcl-2 family, originally identified as an early induction gene during differentiation of myeloid leukemia cells [6]. Mcl-1 contains the Bcl-2 homology (BH) domains BH1-3 and a PEST domain and is a rapidly inducible protein with a short half life [7-9]. It is expressed in various tissues including the liver [10]. In contrast to Bcl-2, Mcl-1 is not only found in mitochondrial membranes, but also in the nucleus and cytoplasm [11]. Several modes of action have been suggested for the anti-apoptotic activity of Mcl-1. Mcl-1 blocks cytochrome *c*-release from mitochondria by interacting with pro-apoptotic members of the Bcl-2 protein family, e.g. Bim [12], Bak [13,14], and NOXA [15]. Furthermore, Mcl-1 interacts with truncated Bid and, thereby, inhibits intrinsic as well as extrinsic apoptotic signaling [16]. Degradation of Mcl-1, e.g. by caspase-3, -8 or granzyme B-mediated cleavage [12], enables proapoptotic Bcl-2 proteins to initiate mitochondrial acitivation.

Mcl-1 has been demonstrated to be highly expressed in various human tumor specimens, e.g. in multiple myeloma, non-small cell lung cancer and liver metastasis of colorectal cancer [17-19]. In addition, Mcl-1 expression correlates with disease grade and survival in human malignancies, e.g. in patients with multiple myeloma or B-cell non-Hodgkin's lymphoma [20,21]. Moreover, Mcl-1 expression predicts response to anti-cancer treatment, e.g. in chronic lymphocytic leukemia or patients with metastasized colorectal cancer [19,22]. Downregulation of Mcl-1 leads to sensitization of tumor cells to different treatment regimens *in vitro*, as shown for cholangiocarcinoma, chronic myelogenous leukemia, sarcoma and malignant melanoma [23-26].

Recently, we and others have shown that Mcl-1 is frequently expressed in tissues of HCC and contributes to apoptosis resistance [27,28]. In non-tumor liver tissue adjacent to HCC Mcl-1 immunoreactivity was significantly lower [27]. No correlation of Mcl-1 expression with the underlying liver disease could be detected [28]. We have also shown that Mcl-1 expression in HCC cells is regulated by different survival pathways such as the PI3K/Akt- and MEK1/Erk-pathway [27].

In this study, we analyze the role of the anti-apoptotic Bcl-2 family member Mcl-1 for the sensitivity of HCC cells towards different treatment regimens such as chemotherapy, kinase inhibition and death receptor ligands. We show that specific downregulation of Mcl-1 by RNA interference leads to significantly higher apoptosis sensitivity of HCC cells. Thus, interference with Mcl-1 expression is an option for the treatment of patients with HCC.

## Methods

### Reagents and cell lines

The human hepatoma cell lines Hep3B and HepG2 were grown in MEM, and Huh7 in DMEM (Invitrogen, Karlsruhe, Germany), all supplemented with 10% fetal bovine serum (Biochrom, Berlin, Germany). Reagents were purchased from the following suppliers: LY294002, PD98059, AG490, Raf I-kinase inhibitor, SU5614 (all solubilized in dimethyl sulfoxide), cisplatin and mitomycin C from Calbiochem (Schwalbach, Germany), valproic acid (VA, orfiril) from Desitin (Hamburg, Germany), 5-Fluorouracil and SP600125 from Sigma (Deisenhofen, Germany).

### Detection of apoptosis

HCC cell lines were seeded onto 12-well plates. On day 3 (PHH) or day 1 (cell lines) after seeding, cells were treated as indicated. After the indicated time periods, cells were collected, washed, and resuspended in lysis buffer containing 0.1% (w/v) sodium citrate, 0.1% (v/v) Triton X-100 and 50 μg/mL propidium iodide (Sigma). After overnight incubation at 4°C, nuclei from apoptotic cells were quantified by flow cytometry according to the method by Nicoletti *et al *[29], using a FACS Calibur (BD Biosciences, Heidelberg, Germany).

### Caspase activities

Cells were lysed in buffer containing 20 mM Tris/HCl pH 8.0, 5 mM EDTA, 0.5% Triton X-100 and 1× complete protease inhibitor cocktail (Roche). Protein concentration was equilibrated by D_c _Protein Assay (Bio-Rad). Lysates were incubated in reaction buffer (25 mM HEPES pH 7.5, 50 mM NaCl, 10% glycerol, 0.05% CHAPS, and 5 mM dithiothreitol) in the presence of 50 μM fluorogenic substrate (Biomol, Germany), specific for by caspase-3 (DEVD-AMC) or caspase-9 (Ac-LEHD-AFC). Assays were performed in black Maxisorb microtiter plates (Nunc, Germany), and the generation of free AMC or AFC at 37°C after 1 h was measured using a fluorometer plate reader (Tecan, Germany) set to an excitation wavelength of 380 nm (AMC and AFC) and an emission wavelength of 460 nm (AMC) or 505 nm (AFC).

### Cell lysis and Western blotting

Cells were lysed by incubation on ice for 15 min in lysis buffer containing 120 mM NaCl, 50 mM Tris/HCl (pH 8.0), 1 % Nonidet P-40, 1 mM phenylmethylsulfonyl fluoride, 25 mM NaF, 0.1% sodium dodecyl sulfate, 100 μM Na_3_VO_4_, 1 mM DTT, and a commercial protease inhibitor cocktail from Roche Diagnostics (Mannheim, Germany). Cell debris was removed by centrifugation (10,000 g; 4°C). Proteins were separated by 10% SDS (sodium dodecyl sulfate)-polyacrylamide gel electrophoresis and transferred to a Hybond ECL nitrocellulose membrane (Amersham Pharmacia Biotech, Freiburg, Germany). Immunodetection was performed using the indicated primary antibodies: anti-Mcl-1 (Santa Cruz Biotechnology, Heidelberg, Germany), mouse anti-alpha-Tubulin clone B-5-1-2 (Sigma). Peroxidase-conjugated antibodies (Santa Cruz Biotechnology) were applied at a concentration of 40 ng/ml. Bound antibody was visualized using chemiluminescent substrate (Perkin-Elmer, Zaventem, Belgium) and exposure to Fuji Medical X-Ray film.

### RNAi for Mcl-1

For small interfering RNA (siRNA)-mediated downregulation of Mcl-1 the following siRNA oligonucleotides were employed (MWG Biotech, Ebersberg, Germany): 5'-aaguaucacagacguucucTT-3' (sense) and 5'-gagaacgucugugauacuuTT-3' (antisense). As a non-silencing control siRNA specific for green fluorescent protein (GFP) was used: 5'-ggcuacguccaggagcgcaccTT-3' (sense) and 5'-ggugcgcuccuggacguagccTT-3' (antisense), where capitals represent DNA-overhangs and lower case letters represent specific RNA-sequences. Huh7 cells were transiently transfected with Transfectin (Bio-Rad, Hercules, CA, USA) according to the manufacturer's protocol and analyzed 24–72 h after transfection.

### Real-Time Quantitative Polymerase Chain Reaction (RT-QPCR)

Total RNA from Huh7 cells was extracted using RNeasy Mini Kit (Qiagen). 1 μg of total RNA was reverse transcribed using an oligo-dT primer and afterwards analyzed by RT-QPCR using the QuantiTect SYBR Green PCR Kit (Qiagen) and the following primers: Actin forward: 5'-GGA CTT CGA GCA AGA GAT GG-3', Actin reverse: 5'-AGC ACT GTG TTG GCG TAC AG-3', Mcl-1 forward: 5'-TAA GGA CAA AAC GGG ACT GG-3', and Mcl-1 reverse: 5'-ACC AGC TCC TAC TCC AGC AA-3'. The relative increase in reporter fluorescent dye emission was monitored. The level of Mcl-1 mRNA, relative to actin, was calculated using the formula: Relative Mcl-1 mRNA expression = 2^[*c*_t_(Mcl-1_control_)-*c*_t_(Mcl-1_treated_)+*c*_t_(Actin_treated_)-*c*_t_(Actin_control_)], where *c*_t _is defined as the number of the cycle in which emission exceeds an arbitrarily defined threshold.

### Statistical analysis

All results are expressed as mean + standard error. Data were analyzed by Student's t test (paired, two sided). P < 0.05 was considered significant.

## Results

### Low sensitivity of Mcl-1 expressing HCC cells towards chemotherapeutic drug-induced apoptosis

**Figure 1:**
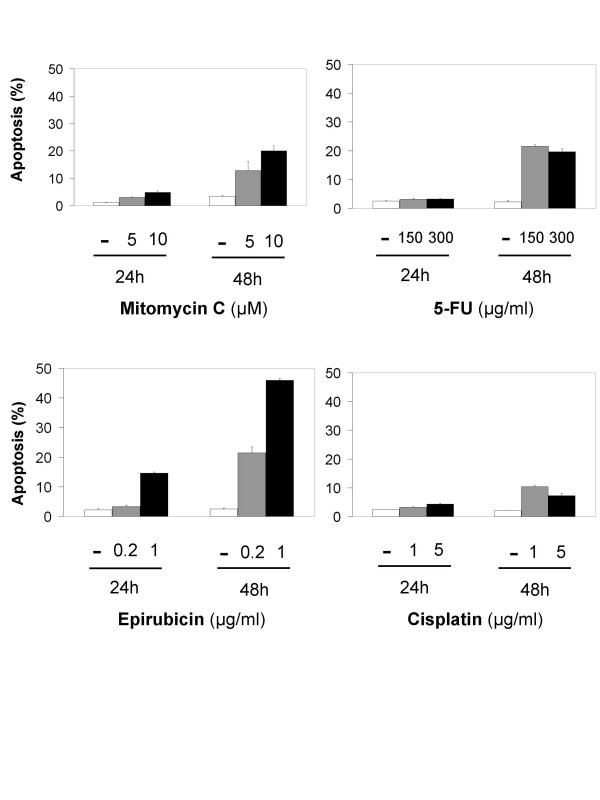
**Sensitivity of Huh7 cells towards chemotherapeutic drug-induced apoptosis**. Huh7 cells were treated with 5-FU, mitomycin C, epirubicin, or cisplatin for 24 h and 48 h, respectively (concentration as indicated). Cells were then harvested and analyzed for apoptosis induction by flow cytometry according to the method of Nicoletti *et al*. [29]. Assays were performed in triplicates and are representative for three independent experiments. Values are means + SD.

Myeloid cell leukemia-1 (Mcl-1) is an important anti-apoptotic factor for HCC [27,28]. We have previously shown that different HCC cell lines such as Huh7, Hep3B and HepG2 show high expression of Mcl-1 [27]. In this study, we first sought to explore the sensitivity of Mcl-1 expressing HCC cell lines to chemotherapeutic drug-induced apoptosis. Huh7, Hep3B and HepG2 cells were treated with different chemotherapeutic drugs, such as mitomycin C and cisplatin (both drugs used in transarterial chemoembolization (TACE) of HCC [30]), epirubicin and 5-Fluorouracil (5-FU) (both used for palliative chemotherapy [31]). In Huh7 cells, treatment with mitomycin C, cisplatin, 5-FU and bleomycin for 24 h resulted in apoptosis rates below 5%. Epirubicin (1 μg/ml), however, led to apoptosis rates of about 15% after 24 h. After treatment for 48 h, 5-FU, bleomycin and mitomycin C induced apoptosis in about 20% of Huh7 cells (5-FU, 150–300 μg/ml, bleomycin, 300 μg/ml, and mitomycin C, 10 μM) (Fig. 1). Cisplatin (1–5 μg/ml) induced apoptosis in only 10% of the cells after 48 h. Comparatively high apoptosis rates were observed after 48 h of epirubicin treatment (46%, 0.2–1 μg/ml) (Fig. 1).

We have already shown in previous studies that Hep3B and HepG2 exhibit only low sensitivity towards treatment with bleomycin and epirubicin [27,32]. In this study, sensitivity of Hep3B and HepG2 cells towards other chemotherapeutic drugs such as 5-FU and cisplatin was comparable to Huh7 (data not shown).

### Enhanced apoptosis sensitivity of Mcl-1 expressing HCC cells to chemotherapy after inhibition of PI3K, but not after inhibition of Jak2, mTOR, MEK1, Src or Raf I kinase

**Figure 2:**
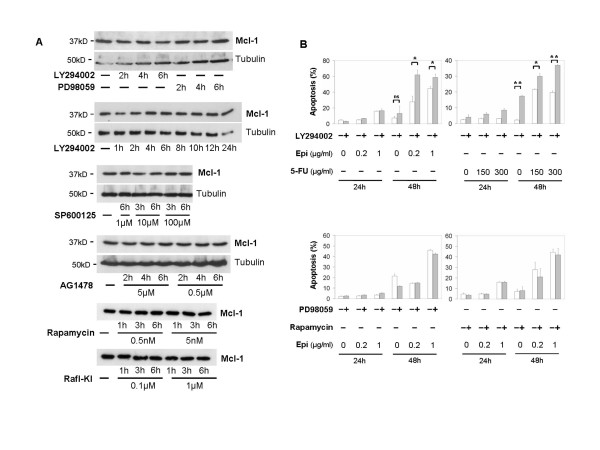
**Blocking of different protein kinases and its effect on Mcl-1 expression and chemotherapeutic drug-induced apoptosis**. (A) Huh7 cells were treated with the kinase inhibitors LY294002 (25 μM, blocking PI3 kinase), PD98059 (50 μM, blocking MEK1), SP600125 (1–100 μM, blocking JNK1), AG1478 (0.5–5 μM, blocking EGF receptor tyrosine kinase), rapamycin (50 pM, blocking mTOR kinase) and Raf I-kinase inhibitor (0.1–1 μM) for the time indicated. Whole cell lysates were prepared, separated, and immunoblotted with antibodies against Mcl-1 and alpha-tubulin. Blots shown are representative for at least two independent experiments. (B) Huh7 cells were treated with LY294002 (25 μM), PD98059 (50 μM), rapamycin (50 pM). After 1 h of pre-incubation, cells were additionally treated with epirubicin, 5-FU or left untreated for 24 h (concentrations as indicated). Cells were then harvested and analyzed by flow cytometry for apoptosis induction according to Nicoletti *et al*. [29]. Assays were performed in triplicates and are representative for three independent experiments. Values are means + SD. *p < 0.01, **p < 0.001.

Next, we analyzed whether interference with Mcl-1 expression could possibly influence the apoptosis sensitivity of HCC cell lines. One approach to influence Mcl-1 expression in liver cells is by blocking kinase signaling pathways. This has been shown for the PI3K/Akt- [33], the MEK1/Erk- [34] and the Jak2/STAT3 signaling pathways [35]. We have already shown that inhibition of PI3 kinase reduced expression of Mcl-1 in Hep3B cells and sensitized cells towards apoptosis induction by bleomycin [27]. In this study we tested the effect of PI3K inhibition on Mcl-1 expression and apoptosis sensitivity in the HCC cell line Huh7. In contrast to Hep3B, even prolonged incubation of Huh7 cells with the PI3K inhibitor LY29004 for up to 24 h did not markedly reduce Mcl-1 expression in Huh7 cells (Fig. 2A). However, after a short incubation for 1 h, a slightly reduced Mcl-1 expression level was observed (Fig. 2A).

PI3K inhibition (LY294002, 50 μM) alone induced apoptosis rates of about 20% after 48 h, and significantly increased chemotherapeutic drug-induced apoptosis: Epirubicin-induced apoptosis was increased from 28 to 62% (0.2 μg/ml, 48 h, p < 0.01) and 5-FU-induced apoptosis from 20 to 37% (300 μg/ml, 48 h, p < 0.001) (Fig. 2B).

In addition, we inhibited other signaling pathways which have been described to influence both the survival of cancer cells and the expression of Bcl-2 family proteins. MEK1/2 is a critical enzyme at the intersection of several biological pathways involved in cancer growth as part of the Ras/Raf/MEK/Erk pathway. In a previous study of our group, inhibition of MEK1 by PD98059 in Hep3B cells neither influenced Mcl-1 expression nor sensitized to bleomycin-induced apoptosis (300 μg/ml, 48 h) [27]. In this study, we also analyzed the effect of MEK1 inhibition on Mcl-1 expression and apoptosis sensitivity in Huh7 cells. As in Hep3B cells, we found that MEK1 inhibition did not influence Mcl-1 expression in these cell lines (Fig. 2A). Neither Mcl-1 expression nor chemotherapeutic drug-induced apoptosis (as tested for cisplatin, epirubicin and 5-FU) was influenced by MEK1 inhibition (Fig. 2A, 2B, and data not shown).

The signaling pathways Jak2/STAT3 as well as Src kinase signaling have been discussed to contribute to apoptosis sensitivity of carcinoma cells including HCC [36-38]. Neither inhibition of Jak2 by AG490 (20 μM, 1–6 h) nor inhibition of Src kinases by PP2 (10 μM, 1–6 h) influenced Mcl-1 expression in Huh7 cells (data not shown). In addition, PP2 (10 μM) and AG490 (20 μM) had no significant impact on 5-FU/VA-induced apoptosis of Huh7 cells (5-FU 150 μg/ml, VA 100 μg/ml, 24 h, data not shown).

Several kinase inhibitors involved in Ras signaling have entered clinical trials [39]. Raf kinase inhibitors such as sorafenib have been proved to exert anti-tumoral activity in patients with advanced HCC [40]. Treatment of Huh7 cells with a Raf I-kinase inhibitor neither altered Mcl-1 expression nor influenced sensitivity towards chemotherapeutic drug-induced apoptosis (Fig. 2A, and data not shown). The mTOR protein kinase has emerged as a critical growth-control kinase and receives stimulatory signals from Ras and other kinases. mTOR-inhibition showed anti-tumoral activity in HCC patients [41]. The widely used mTOR inhibitor rapamycin did not influence Mcl-1 expression or apoptosis sensitivity of Huh7 cells (Fig. 2A, 2B).

### Sensitizing HCC cells towards chemotherapeutic drug-induced apoptosis by RNAi of Mcl-1 expression

**Figure 3:**
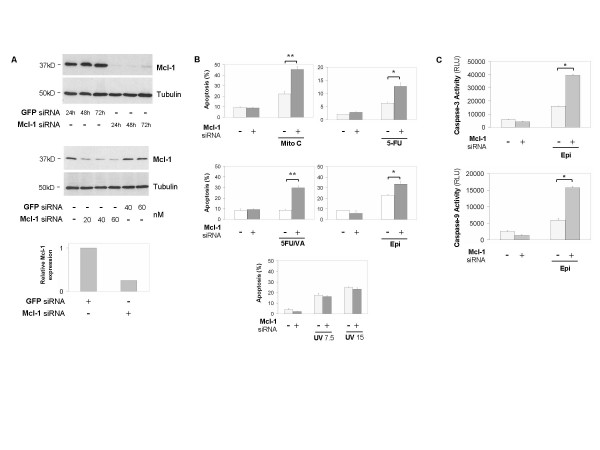
**Silencing of Mcl-1 expression in HCC cells by RNA interference results in sensitization towards chemotherapy**. (A) Huh7 cells were transfected with siRNA specific for Mcl-1 (40 nM) or transfected with siRNA specific for green fluorescent protein (GFP) as control. Cells were lysed 24, 48 and 72 h after transfection and analyzed for Mcl-1 expression by Western Blot (upper panel). Tubulin expression was used to control equal loading. siRNA concentration was titrated as indicated and expression of Mcl-1 and tubulin was analyzed by Western Blot 24 h after transfection (middle panel). 24 h after transfection with siRNA (40 nM), total RNA was extracted and analyzed for Mcl-1 mRNA expression by quantitative real-time PCR (lower panel). Expression of Mcl-1 was normalized to actin in each sample. Relative Mcl-1 expression was calculated as described in the materials and methods section. (B) Huh7 cells were transfected with siRNA specific for Mcl-1 (40 nM, "Mcl-1 siRNA +") or transfected with siRNA specific for GFP as control ("Mcl-1 siRNA -"). 24 h after transfection cells were treated with different chemotherapeutic drugs for 24 h (mitomycin C, 10 μM, 5-FU, 150 μg/ml (with or without valproic acid, VA, 100 μg/ml) and epirubicin, 1 μg/ml). Some cultures were irradiated with UV (7.5 and 15 mJ/cm^2^, respectively). 24 h later, cells were harvested and analyzed by flow cytometry for apoptosis induction according to the method by Nicoletti [29]. Assays were performed in triplicates and are representative for at least three independent experiments. Values are means + SD. *p < 0.01, **p < 0.001. (C) Cells were incubated for 24 h with epirubicin (1 μg/ml). Caspase-3 (upper panel) and caspase-9 (lower panel) activity were measured by fluorometric assays as described in the materials and methods section. Bars = SD.

HCC cells are rather resistant to chemotherapeutic drug-induced apoptosis. The Bcl-2 family member Mcl-1 has been described to contribute to the resistant phenotype in hepatocellular carcinoma [27,28]. Thus, a potential approach to sensitize HCC cells to chemotherapy is the specific knock down of Mcl-1. In this study, we applied RNA interference to downregulate Mcl-1 expression in the HCC cell line Huh7. Transfection with Mcl-1 specific siRNA led to a profound decrease of Mcl-1 expression on mRNA as well as protein level already 24 h after transfection and continues for at least 72 h (Fig. 3A). Next, we tested the effect of Mcl-1 downregulation on chemotherapeutic drug-induced apoptosis of HCC cells. Mcl-1 downregulation by siRNA alone did not induce apoptosis (Fig. 3B). However, Mcl-1 downregulation sensitized Huh7 cells towards a panel of chemotherapeutic drugs including epirubicin, mitomycin C, and 5-FU (Fig. 3B). For example, epirubicin-induced apoptosis was enhanced from 23 to 34% (1 μg/ml, 24 h, p < 0.01) and mitomycin C-induced apoptosis from 22% to 46% (10 μM, 24 h, p < 0.001). In a previous study, we have shown that 5-FU-induced apoptosis of hepatoma cells is increased by addition of histone deacetylase inhibitors such as valproic acid (VA) [32]. 5-FU/VA-induced apoptosis of Huh7 cells was increased from 9 to 30% by knockdown of Mcl-1 (5-FU 150 μg/ml, VA 100 μg/ml, 24 h, p < 0.001) (Fig. 3B). Application of siRNA specific for Mcl-1 only slightly enhanced cisplatin-induced apoptosis compared to cells transfected with control siRNA (data not shown). Furthermore, downregulation of Mcl-1 did not sensitize Huh7 cells towards UV irradiation-induced apoptosis (7.5 and 15 mJ/cm^2^, 24 h; Fig. 3B).

Cleavage and activation of caspases is a central event in apoptosis pathways. Thus, we also tested the activity of caspase-3 and -9 after chemotherapy with or without downregulation of Mcl-1. In cells with knockdown of Mcl-1, caspase activity was profoundly higher after treatment with chemotherapeutics compared to mock treated cells. Treatment with epirubicin (1 μg/ml, 24 h), for example, resulted in significant higher caspase-3 and -9 activities compared to epirubicin-treated control cells (Fig. 3C).

### Sensitizing HCC cells towards PI3K by specific downregulation of Mcl-1

Targeted therapy by kinase inhibitors, such as the Raf kinase inhibitor sorafenib, is already applied in clinical trials for the treatment of advanced hepatocellular carcinoma [40]. In this study, we analyzed the relevance of Mcl-1 expression for apoptosis induced by different kinase inhibitors.

**Figure 4:**
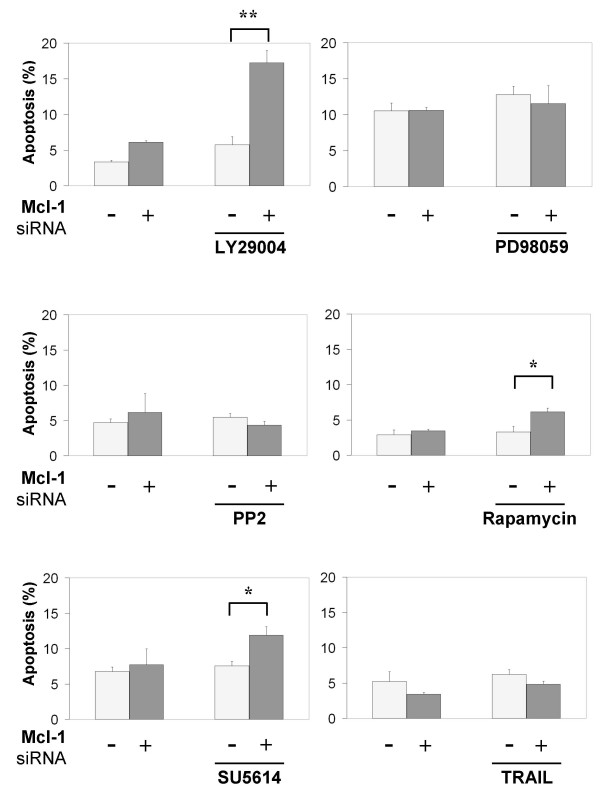
**Apoptosis sensitivity of HCC cells towards different kinase inhibitors and TRAIL after downregulation of Mcl-1**. Huh7 cells were treated for 24 h with different kinase inhibitors (LY294002, 25 μM; PD98059, 50 μM; PP2, 10 μM; rapamycin, 50 pM; SU5614, 15 μM; and TRAIL, 100 ng/ml). Cells were transfected 24 h before with siRNA specific for Mcl-1 (40 nM, "Mcl-1 siRNA +") or with siRNA specific for green fluorescent protein as a control (40 nM, "Mcl-1 siRNA -"). Apoptosis was determined by FACS analysis according to Nicoletti [29]. Values are means + SD. *p < 0.01, **p < 0.001.

**Figure 5:**
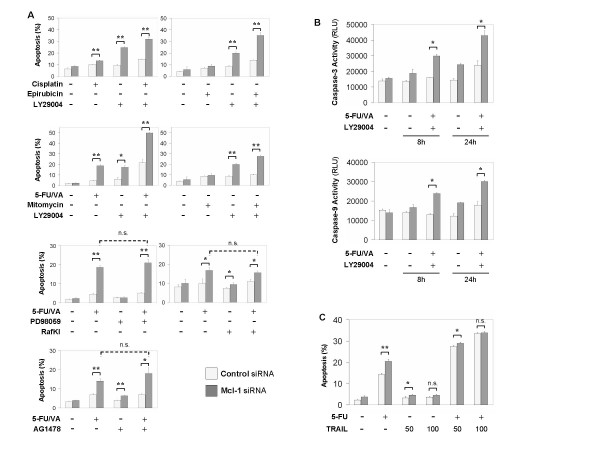
**Mcl-1 interference sensitizes HCC cells towards combined treatment modalities**. (A) Huh7 cells were transfected with siRNA specific for Mcl-1 (grey bars) or transfected with siRNA specific for GFP as control (light bars). After 24 h, cells were treated with chemotherapeutic drugs for another 24 h: cisplatin, 1 μg/ml, epirubicin, 200 ng/ml, 5-FU, 150 μg/ml (+ valproic acid (VA), 100 μg/ml), and mitomycin C, 5 μM. Some cultures were pre-incubated for 1 h with the kinase inhibitors LY294002 (25 μM, blocking PI3K), PD98059 (50 μM, blocking MEK1), AG1478 (5 μM, blocking EGF receptor tyrosine kinase) and a Raf I-kinase inhibitor (100 nM). Apoptosis was assessed by FACS analysis. *p < 0.05, **p < 0.001. n.s., not significant. (B) and (C) Huh7 cells were transfected with siRNA (40 nM) as described above and treated 24 h thereafter. In (B), cells were treated with 5-FU (150 μg/ml) and VA (100 μg/ml) for 8 or 24 h, as indicated. Cells were harvested and caspase-3 (upper panel) or caspase-9 activities (lower panel) were measured by applying caspase-specific fluorogenic substrates as described in the materials and methods section. *p < 0.01, **p < 0.001. (C) Cells were treated with 5-FU (150 μg/ml) for 48 h with or without simultaneous treatment with TRAIL as indicated. Apoptosis was measured using flow cytometrical analysis. Values are means + SD. *p < 0.05, **p < 0.001, n.s., not significant.

Inhibition of PI3 kinase in Huh7 cells by LY294002 alone induced apoptosis only to a minor extent (Fig. 4). However, after specific downregulation of Mcl-1 by RNA interference, apoptosis was induced (17.2% after 24 h, p < 0.001) (Fig. 4). In contrast, neither MEK1 inhibition by PD98059 (50 μM, 24 h), nor inhibition of Src kinases by PP2 (10 μM, 24 h) induced apoptosis in cells with downregulated Mcl-1 (Fig. 4). In contrary, knockdown of Mcl-1 slightly sensitized HCC cells towards inhibition of mTOR by rapamycin (5 nM, 24 h, p < 0.01), selective inhibition of VEGF (Flk-1) and PDGF receptor tyrosine kinases by SU5614 (15 μM, 24 h, p < 0.01) (Fig. 4) and inhibition of the Raf I kinase (p < 0.05, 100 nM, 24 h, Fig. 5A).

Epidermal Growth Factor (EGF) is involved in carcinogenesis and apoptosis sensitivity of cancer cells. Inhibition of EGF receptor tyrosine kinase activity by AG1478 (5 μM, 24 h), slightly sensitized cells towards Mcl-1 downregulation (p < 0.001, Fig. 5A).

Tumor necrosis factor-related apoptosis-inducing ligand (TRAIL/Apo2L) exhibits potent antitumor activity on systemic administration and is potentially safe to hepatocytes in a monotherapeutic approach [42]. Treatment of Huh7 cells with TRAIL alone (100 ng/ml, 24 h) did not induce apoptosis. In addition, no sensitization was observed by downregulation of Mcl-1 (Fig. 4). However, in a concentration of 50 ng/ml, TRAIL-induced apoptosis was slightly enhanced by downregulation of Mcl-1 (Fig. 5A, p < 0.05).

### Sensitizing HCC cells towards combined treatment modalities after downregulation of Mcl-1

One approach to treat advanced HCC is the combined application of chemotherapy and protein kinase inhibitors or cytotoxic antibodies [43]. Thus, we tested whether the effectiveness of combined treatment regimens can be increased by knockdown of Mcl-1. Knockdown of Mcl-1 resulted in enhanced apoptosis induction in Huh7 cells treated with the PI3K inhibitor LY294002 and chemotherapeutics. For example, apoptosis induction by treatment with LY294002 (25 μM) and cisplatin (1 μg/ml, 24 h) was enhanced from 15% (cells transfected with control siRNA) to 32% (cells transfected with Mcl-1 siRNA, p < 0.001). The sensitizing effect of Mcl-1 RNA interference was observed in combination with all chemotherapeutic agents tested in this setting (epirubicin, 5-FU/VA, mitomycin C and cisplatin) (Fig. 5A). Additionally, Mcl-1 downregulation sensitized cells towards chemotherapy combined with MEK1, Raf I kinase, mTOR, VEFG/PDGF receptor tyrosine kinase, or EGF receptor tyrosine kinase inhibition (Fig. 5A, and data not shown). However, apoptosis rates were not significantly increased by these inhibitors compared to Mcl-1 downregulation and chemotherapy alone.

No sensitization by Mcl-1 downregulation was observed in cells treated with 5-FU/VA and the JNK1-inhibitor SP600125 or the Src kinase inhibitor PP2, respectively (data not shown). Sensitization of HCC cells towards treatment-induced apoptosis was accompanied by enhanced activities of caspase-3 and -9. For example, in cells co-treated with 5-FU (150 μg/ml), VA (100 μg/ml) and LY294002 (25 μM) for 24 h, caspase-3 activity was about 30,000 arbitrary units after 8 h and about 43,000 after 24 h in cells treated with Mcl-1 siRNA vs. 16,000 after 8 h and 24,000 after 24 h in cells treated with control siRNA (Fig. 5B).

Pre-clinical studies proved that co-treatment of TRAIL and chemotherapeutic drugs can overcome resistance to chemotherapy in many cancer types including HCC. Chemotherapy sensitizes HCC cells to TRAIL partly via activation of the death inducing signaling complex [44]. In the current study, Mcl-1 downregulation slightly sensitized Huh7 cells towards co-treatment with TRAIL and 5-FU (50 ng/ml and 150 μg/ml, 24 h, 28.9 vs. 27.4% apoptosis, p < 0.05, Fig. 5C).

## Discussion

Therapy resistance is a common clinical problem in hepatocellular carcinoma (HCC). In the current study we applied RNA interference to specifically downregulate the anti-apoptotic Bcl-2 protein Mcl-1 in HCC cells to overcome resistance. After Mcl-1 knockdown, HCC cells proved to be more sensitive towards apoptosis induction by chemotherapy and molecularly targeted therapy. Our data suggest that Mcl-1 is a promising target for therapeutic approaches in patients with HCC. Since transgenic deletion of Mcl-1 in the liver *in vivo *does not induce apoptosis in normal hepatocytes, targeting of Mcl-1 in HCC cells is likely to be tolerated by the surrounding liver tissue [45].

HCC is considered highly resistant to chemotherapy. This is in part due to a high expression rate of drug resistance genes, including p-glycoprotein, glutathione-S-transferase, heat shock proteins, and mutations in p53. Additionally, resistance to apoptosis is a principal mechanism through which HCC cells are enabled to survive therapy, since chemotherapy and irradiation kill tumor cells mainly by induction of apoptosis [46]. In this study, we first tested the sensitivity of Mcl-1 expressing HCC cells to a panel of chemotherapeutic drugs. Mitomycin C, 5-FU and bleomycin treatment of different HCC cell lines only induced low apoptosis rates (below 5% after 24 h; 10–20% after 48 h). Cisplatin is widely administered locally and systemically in the treatment of advanced HCC [30]. However, apoptosis was induced in only 10% of the HCC cell lines after treatment with cisplatin for 48 h. The anthracycline derivative epirubicin induced higher apoptosis rates (about 45% after 48 h). In line with this, anthracyclines are currently among the most effective chemotherapeutic agents against HCC and the most frequently used anticancer drugs for monosystemic treatment of advanced hepatocellular carcinoma [31,47]. However, systemic treatment with anthracyclines alone mostly failed to demonstrate any survival benefit for HCC patients [48]. Thus, combination chemotherapy regimens have been tested or are currently in clinical trials in patients with advanced HCC. So far, median survival in all of these studies has been short despite objective responses.

Several defects in apoptosis signaling cells have been discussed in the past conferring drug resistance of HCC. These defects include, among others, stabilization of mitochondria, alterations in survival signaling and inactivation of death receptor signaling [5]. Anti-apoptotic members of the Bcl-2 family, such as Bcl-2, Bcl-x_L_, Bfl-1 and Mcl-1, critically regulate the integrity of mitochondria and have been shown to prevent apoptosis by anticancer drugs *in vitro *[4]. Enhanced expression of anti-apoptotic Bcl-2 proteins is frequently observed in malignancies of diverse origin, e.g. Bfl-1 in diffuse large-cell lymphoma [49] and Bcl-x_L _in lung adenocarcinoma [50]. Pharmacological manipulation of Bcl-2 family members appears therefore efficient in cancer treatment [51].

We have previously shown that Mcl-1 expression is considerably enhanced in human HCC tissue compared to adjacent non-tumor tissue [27]. Increased Mcl-1 expression has already been demonstrated for other malignancies, e.g. multiple myeloma and non-small cell lung cancer [17,18]. Mcl-1 significantly correlates with Bcl-x_L _expression in HCC tissue [28]. More studies are required to fully understand the individual roles of the Bcl-2 proteins and how they cooperate to regulate HCC cell survival.

Our results demonstrate that a specific knockdown of Mcl-1 by RNAi sensitizes HCC cells to chemotherapeutic drugs, such as epirubicin, mitomycin C and 5-FU. These results extend studies on hematopoietic cells which demonstrate that Mcl-1 prolongs survival after exposure to chemotherapeutic drugs [52]. Our data also add to studies on other tumor types such as malignant melanoma and sarcoma, in which specific downregulation of Mcl-1 has been shown to sensitize cancer cells to chemotherapeutic drug-induced apoptosis [25,26].

Direct targeting of Mcl-1 by antisense oligonucleotides has already been shown to sensitize the HCC cell line HepG2 as well as lung carcinoma cell lines to cisplatin-induced apoptosis [18,28]. However, in Huh7 cells, we did not observe enhanced cisplatin-induced apoptosis upon depletion of Mcl-1. This effect might be cell line- specific.

Mcl-1 knockdown also augmented tumor cell death after inhibition of the pro-survival PI3K/Akt pathway. This pathway contributes to apoptosis resistance in various malignancies. Active PI3K generates the phosphorylated lipid phosphatidylinositol-3,4,5-triphosphate (PtdIns(3,4,5)P3), leading to the recruitment and activation of other kinases such as Akt (protein kinase B) to the plasma membrane. Activated Akt induces strong cellular survival signals. We have already shown that the PI3K/Akt pathway induces Mcl-1 expression in human hepatocytes [33]. In the current study, PI3K inhibition for 48 h led to apoptosis rates of about 20%. Downregulation of Mcl-1 significantly enhanced apoptosis induced by PI3K inhibition. Furthermore, Mcl-1 downregulation further augmented apoptosis caused by combination therapy with PI3K inhibition and chemotherapeutics.

One of the downstream targets of Akt is the serine/threonine kinase mTOR which is a promising candidate target in the PI3K/Akt pathway in HCC [43]. In the current study, Mcl-1 downregulation slightly enhanced apoptosis induction in cells treated with chemotherapy and rapamycin. However, this treatment was not superior to the combination of Mcl-1 downregulation and chemotherapy without rapamycin.

The Ras/Raf/MEK/ERK pathway is another critical signaling cascade in HCC [38,53]. Approaches to disable the MEK1/ERK pathway may also sensitize tumor cells to apoptosis induction. ERK phosphorylates and thereby activates downstream targets like the transcription factor c-Jun. In the current study, MEK1 inhibition by PD98059 with or without downregulation of Mcl-1 did not induce significant apoptosis rates in Huh7 cells. Mcl-1 downregulation, however, when combined with MEK1 inhibition and chemotherapy, triggered apoptosis in Huh7 cells. This treatment, however, did not induce higher apoptosis rates than Mcl-1 downregulation and chemotherapy alone.

Mcl-1 downregulation also enhanced apoptosis induction in cells treated with chemotherapy and Raf I kinase inhibition. Again, apoptosis rates were not higher than after treatment with Mcl-1 downregulation and chemotherapy alone. Thus, it still remains elusive, if the combination of chemotherapy, Mcl-1 RNAi and inhibitors of the Ras/Raf/MEK/ERK pathway is more efficient than the treatment with chemotherapy and Mcl-1 RNAi alone.

Targeting receptor tyrosine kinases (RTKs) with small molecule inhibitors recently emerged as a compelling new approach to cancer therapy. RTKs are involved in the formation and progression of solid tumors such as HCC. Many kinase inhibitors currently in clinical trials target RTKs. For example, EGFR tyrosine kinase inhibitors are tested in clinical trials in patients with advanced HCC [54]. HCC tissues show significant expression of EGFR [54]. In the current study, EGFR tyrosine kinase inhibition alone did not result in HCC cell death even if combined with Mcl-1 downregulation. However, combined treatment with Mcl-1 siRNA, 5-FU/valproic acid (VA) and the EGFR tyrosine kinase inhibitor AG1478 resulted in more than two-fold enhanced apoptosis rates compared to treatment with control siRNA, 5-FU/VA and AG1478. Noteworthy, however, the combined treatment was not superior to the combination of Mcl-1 siRNA and chemotherapy alone.

VEGF receptor tyrosine kinase (VEGFR) is another promising target for the treatment of HCC. HCCs are vascularized tumors with high expression of VEGF raising the possibility that agents targeting VEGFR might be of therapeutic value. In this study, Mcl-1 knockdown sensitized HCC cells to the VEGF/PDGF inhibitor SU5614. Mcl-1 downregulation might improve therapeutic regimens targeting VEGF or PDGF signaling in HCC. No sensitizing effect of Mcl-1 knockdown in HCC cells was observed for the treatment with JNK1 and Src kinase inhibitors. The same applied for Jak2 inhibitors, although Jak2 has been shown to be ubiquitously activated in human HCC [38].

The death receptor ligand TRAIL is another promising anti-cancer agent. We have previously shown that treatment with TRAIL alone or in combination with chemotherapeutic drugs (with the exception of cisplatin) is not toxic for human hepatocytes [42]. Thus, treatment with TRAIL and chemotherapeutic drugs is a potential therapeutic approach to treatment of HCC. Mcl-1 downregulation has already been shown to sensitize cholangiocarcinoma cells to TRAIL-induced apoptosis [23] and CML cells towards treatment with imatinib [24]. We did not observe significant apoptosis rates in Huh7 treated with recombinant TRAIL and Mcl-1 siRNA. However, cells with downregulated Mcl-1 showed slightly higher apoptosis rates after treatment with TRAIL (significant difference for TRAIL 50 ng/ml). In line with previous results, 5-FU and TRAIL together induced apoptosis in Huh7 cells [44]. Again, Mcl-1 downregulation only sligthly sensitized Huh7 cells to this combinatorial approach (significant difference for 50 ng/ml).

In this study we applied RNA interference (RNAi) to knock down Mcl-1 expression. RNAi is a phenomenon in which genes are specifically silenced at the level of mRNA degradation [55]. In the current study, transfection of chemically synthesized siRNA had a fast inhibitory effect on Mcl-1 expression in HCC cells *in vitro*. 24 h after transfection of HCC cells with siRNA, Mcl-1 expression was profoundly reduced. siRNA can also be applied in animals *in vivo *by intravenous or local injection. For the use *in vivo*, the transit of siRNA to the target cells, e.g. HCC cells, is a major obstacle. However, RNAi-based approaches have already shown promising preclinical results in animal models [56]. In a mouse model of fulminant hepatitis, intravenous application of siRNA targeting the death receptor CD95 (APO-1/Fas) was capable of preventing liver injury [57].

An alternative approach to selectively downregulate Mcl-1 is the application of antisense oligonucleotides (ASO). ASO are chemically modified stretches of single stranded DNA designed to bind to a specific mRNA and selectively suppress its translation. ASOs have already entered phase III clinical trails for the modulation of the chemosensitivity of human malignancies. Antisense strategies targeting anti-apoptotic Bcl-2 family proteins including Mcl-1 have been successfully applied in various human malignancies *in vitro *and *in vivo *[25,58,59]. In contrast to the results of the current study, Mcl-1 downregulation by ASO treatment in HCC cell lines resulted in spontaneous apoptosis without an additional apoptotic stimulus [28]. The reason for this is elusive. It is not likely due to a more effective knockdown of Mcl-1 by ASO application: 24 to 72 h after transfection with Mcl-1 siRNA, virtually no Mcl-1 expression could be detected by Western blot analysis in our study. One reason might be the different identity of the HCC cell lines analyzed in both studies.

The molecular mechanisms that mediate the pro-apoptotic effect of Mcl-1 downregulation in HCC cells remain elusive and are subject to further studies. An important mode of action of Mcl-1 is the interaction with pro-apoptotic Bcl-2 family members (such as Bim [60], Bak, Bid and Bax). Heterodimerization of Mcl-1 with proapoptotic Bcl-2 family members can neutralize their proapoptotic properties. After knockdown of Mcl-1 by RNAi, levels of free Bim might be increased which in turn may lead to activation of Bax. The importance of the Mcl-1-Bim complex for apoptotic signaling has been demonstrated previously [60]. Elevated levels of free Bim might sensitize cells to apoptotic stimuli such as chemotherapy. Another mechanism which would explain the sensitizing effects of Mcl-1 knockdown would be the release of Bak. Mcl-1 is known to interact with Bak [13]. This complex can be disrupted by p53. However, since p53 mutations are frequently found in HCC, Mcl-1-Bak interaction might be more stable in HCC, resulting in a stabilization of mitochondria. This might also apply for the p53-/- cell line Huh7, which we used as a model system for silencing Mcl-1. However, other mechanisms such as the induction of Ca2+-signaling by elimination of Mcl-1 (as shown in a previous study [60]), might also explain the sensitizing effects of Mcl-1 RNAi.

Numerous genes may provide attractive targets for RNAi in patients with HCC. This study demonstrates that targeting of Mcl-1 by siRNA sensitizes HCC cell lines to chemotherapy and molecularly targeted therapy. In the future it may be promising to target more than one gene involved in apoptosis signaling, e.g. by a mixture of siRNAs or by the use of plasmids expressing a number of shRNAs [56].

## Conclusion

Our study supports the concept that Mcl-1 is an important survival factor for hepatocellular carcinoma. Targeting of Mcl-1 via RNA interference is a potential therapeutic strategy to render HCC cells more sensitive to chemotherapy and molecularly targeted treatment regimens.

## Competing interests

The author(s) declare that they have no competing interests.

## Authors' contributions

HSB and BF were performing the experiments of the study and made substantial contributions to conception and design of the study, interpretation of the data and statistical analysis. HSB drafted the manuscript. PK, A. Weber, MS and PRG made substantial contributions to conception and design and interpretation of data. A. Weinmann and MS participated in the design of the study and in data analyses. All authors read and approved the final manuscript.

## Pre-publication history

The pre-publication history for this paper can be accessed here:



## References

[B1] Llovet JM, Burroughs A, Bruix J (2003). Hepatocellular carcinoma. Lancet.

[B2] Bruix J, Llovet JM (2002). Prognostic prediction and treatment strategy in hepatocellular carcinoma. Hepatology.

[B3] Bruix J, Sherman M (2005). Management of hepatocellular carcinoma. Hepatology.

[B4] Kroemer G, Reed JC (2000). Mitochondrial control of cell death. Nat Med.

[B5] Schulze-Bergkamen H, Krammer PH (2004). Apoptosis in cancer--implications for therapy. Semin Oncol.

[B6] Kozopas KM, Yang T, Buchan HL, Zhou P, Craig RW (1993). MCL1, a gene expressed in programmed myeloid cell differentiation, has sequence similarity to BCL2. Proc Natl Acad Sci U S A.

[B7] Akgul C, Turner PC, White MR, Edwards SW (2000). Functional analysis of the human MCL-1 gene. Cell Mol Life Sci.

[B8] Schubert KM, Duronio V (2001). Distinct roles for extracellular-signal-regulated protein kinase (ERK) mitogen-activated protein kinases and phosphatidylinositol 3-kinase in the regulation of Mcl-1 synthesis. Biochem J.

[B9] Craig RW (2002). MCL1 provides a window on the role of the BCL2 family in cell proliferation, differentiation and tumorigenesis. Leukemia.

[B10] Krajewski S, Bodrug S, Krajewska M, Shabaik A, Gascoyne R, Berean K, Reed JC (1995). Immunohistochemical analysis of Mcl-1 protein in human tissues. Differential regulation of Mcl-1 and Bcl-2 protein production suggests a unique role for Mcl-1 in control of programmed cell death in vivo. Am J Pathol.

[B11] Yang T, Kozopas KM, Craig RW (1995). The intracellular distribution and pattern of expression of Mcl-1 overlap with, but are not identical to, those of Bcl-2. J Cell Biol.

[B12] Han J, Goldstein LA, Gastman BR, Rabinovitz A, Rabinowich H (2005). Disruption of Mcl-1.Bim complex in granzyme B-mediated mitochondrial apoptosis. J Biol Chem.

[B13] Leu JI, Dumont P, Hafey M, Murphy ME, George DL (2004). Mitochondrial p53 activates Bak and causes disruption of a Bak-Mcl1 complex. Nat Cell Biol.

[B14] Willis SN, Chen L, Dewson G, Wei A, Naik E, Fletcher JI, Adams JM, Huang DC (2005). Proapoptotic Bak is sequestered by Mcl-1 and Bcl-xL, but not Bcl-2, until displaced by BH3-only proteins. Genes Dev.

[B15] Chen L, Willis SN, Wei A, Smith BJ, Fletcher JI, Hinds MG, Colman PM, Day CL, Adams JM, Huang DC (2005). Differential targeting of prosurvival Bcl-2 proteins by their BH3-only ligands allows complementary apoptotic function. Mol Cell.

[B16] Clohessy JG, Zhuang J, de Boer J, Gil-Gomez G, Brady HJ (2006). Mcl-1 interacts with truncated Bid and inhibits its induction of cytochrome c release and its role in receptor-mediated apoptosis. J Biol Chem.

[B17] Derenne S, Monia B, Dean NM, Taylor JK, Rapp MJ, Harousseau JL, Bataille R, Amiot M (2002). Antisense strategy shows that Mcl-1 rather than Bcl-2 or Bcl-x(L) is an essential survival protein of human myeloma cells. Blood.

[B18] Song L, Coppola D, Livingston S, Cress D, Haura EB (2005). Mcl-1 regulates survival and sensitivity to diverse apoptotic stimuli in human non-small cell lung cancer cells. Cancer Biol Ther.

[B19] Backus HH, van Riel JM, van Groeningen CJ, Vos W, Dukers DF, Bloemena E, Wouters D, Pinedo HM, Peters GJ (2001). Rb, mcl-1 and p53 expression correlate with clinical outcome in patients with liver metastases from colorectal cancer. Ann Oncol.

[B20] Wuilleme-Toumi S, Robillard N, Gomez P, Moreau P, Le Gouill S, Avet-Loiseau H, Harousseau JL, Amiot M, Bataille R (2005). Mcl-1 is overexpressed in multiple myeloma and associated with relapse and shorter survival. Leukemia.

[B21] Cho-Vega JH, Rassidakis GZ, Admirand JH, Oyarzo M, Ramalingam P, Paraguya A, McDonnell TJ, Amin HM, Medeiros LJ (2004). MCL-1 expression in B-cell non-Hodgkin's lymphomas. Hum Pathol.

[B22] Saxena A, Viswanathan S, Moshynska O, Tandon P, Sankaran K, Sheridan DP (2004). Mcl-1 and Bcl-2/Bax ratio are associated with treatment response but not with Rai stage in B-cell chronic lymphocytic leukemia. Am J Hematol.

[B23] Taniai M, Grambihler A, Higuchi H, Werneburg N, Bronk SF, Farrugia DJ, Kaufmann SH, Gores GJ (2004). Mcl-1 mediates tumor necrosis factor-related apoptosis-inducing ligand resistance in human cholangiocarcinoma cells. Cancer Res.

[B24] Aichberger KJ, Mayerhofer M, Krauth MT, Skvara H, Florian S, Sonneck K, Akgul C, Derdak S, Pickl WF, Wacheck V, Selzer E, Monia BP, Moriggl R, Valent P, Sillaber C (2005). Identification of mcl-1 as a BCR/ABL-dependent target in chronic myeloid leukemia (CML): evidence for cooperative antileukemic effects of imatinib and mcl-1 antisense oligonucleotides. Blood.

[B25] Thallinger C, Wolschek MF, Maierhofer H, Skvara H, Pehamberger H, Monia BP, Jansen B, Wacheck V, Selzer E (2004). Mcl-1 is a novel therapeutic target for human sarcoma: synergistic inhibition of human sarcoma xenotransplants by a combination of mcl-1 antisense oligonucleotides with low-dose cyclophosphamide. Clin Cancer Res.

[B26] Thallinger C, Wolschek MF, Wacheck V, Maierhofer H, Gunsberg P, Polterauer P, Pehamberger H, Monia BP, Selzer E, Wolff K, Jansen B (2003). Mcl-1 antisense therapy chemosensitizes human melanoma in a SCID mouse xenotransplantation model. J Invest Dermatol.

[B27] Fleischer B, Schulze-Bergkamen H, Schuchmann M, Weber A, Biesterfeld S, Muller M, Krammer PH, Galle PR (2006). Mcl-1 is an anti-apoptotic factor for human hepatocellular carcinoma. Int J Oncol.

[B28] Sieghart W, Losert D, Strommer S, Cejka D, Schmid K, Rasoul-Rockenschaub S, Bodingbauer M, Crevenna R, Monia BP, Peck-Radosavljevic M, Wacheck V (2006). Mcl-1 overexpression in hepatocellular carcinoma: a potential target for antisense therapy. J Hepatol.

[B29] Nicoletti I, Migliorati G, Pagliacci MC, Grignani F, Riccardi C (1991). A rapid and simple method for measuring thymocyte apoptosis by propidium iodide staining and flow cytometry. J Immunol Methods.

[B30] Burroughs A, Hochhauser D, Meyer T (2004). Systemic treatment and liver transplantation for hepatocellular carcinoma: two ends of the therapeutic spectrum. Lancet Oncol.

[B31] Pohl J, Zuna I, Stremmel W, Rudi J (2001). Systemic chemotherapy with epirubicin for treatment of advanced or multifocal hepatocellular carcinoma. Chemotherapy.

[B32] Schuchmann M, Schulze-Bergkamen H, Fleischer B, Schattenberg JM, Siebler J, Weinmann A, Teufel A, Worns M, Fischer T, Strand S, Lohse AW, Galle PR (2006). Histone deacetylase inhibition by valproic acid down-regulates c-FLIP/CASH and sensitizes hepatoma cells towards CD95- and TRAIL receptor-mediated apoptosis and chemotherapy. Oncol Rep.

[B33] Schulze-Bergkamen H, Brenner D, Krueger A, Suess D, Fas SC, Frey CR, Dax A, Zink D, Buchler P, Muller M, Krammer PH (2004). Hepatocyte growth factor induces Mcl-1 in primary human hepatocytes and inhibits CD95-mediated apoptosis via Akt. Hepatology.

[B34] Huang HM, Huang CJ, Yen JJ (2000). Mcl-1 is a common target of stem cell factor and interleukin-5 for apoptosis prevention activity via MEK/MAPK and PI-3K/Akt pathways. Blood.

[B35] Liu H, Ma Y, Cole SM, Zander C, Chen KH, Karras J, Pope RM (2003). Serine phosphorylation of STAT3 is essential for Mcl-1 expression and macrophage survival. Blood.

[B36] Yoshikawa H, Matsubara K, Qian GS, Jackson P, Groopman JD, Manning JE, Harris CC, Herman JG (2001). SOCS-1, a negative regulator of the JAK/STAT pathway, is silenced by methylation in human hepatocellular carcinoma and shows growth-suppression activity. Nat Genet.

[B37] Park SS, Eom YW, Kim EH, Lee JH, Min do S, Kim S, Kim SJ, Choi KS (2004). Involvement of c-Src kinase in the regulation of TGF-beta1-induced apoptosis. Oncogene.

[B38] Calvisi DF, Ladu S, Gorden A, Farina M, Conner EA, Lee JS, Factor VM, Thorgeirsson SS (2006). Ubiquitous activation of Ras and Jak/Stat pathways in human HCC. Gastroenterology.

[B39] Sebolt-Leopold JS, Dudley DT, Herrera R, Van Becelaere K, Wiland A, Gowan RC, Tecle H, Barrett SD, Bridges A, Przybranowski S, Leopold WR, Saltiel AR (1999). Blockade of the MAP kinase pathway suppresses growth of colon tumors in vivo. Nat Med.

[B40] Strumberg D, Richly H, Hilger RA, Schleucher N, Korfee S, Tewes M, Faghih M, Brendel E, Voliotis D, Haase CG, Schwartz B, Awada A, Voigtmann R, Scheulen ME, Seeber S (2005). Phase I clinical and pharmacokinetic study of the Novel Raf kinase and vascular endothelial growth factor receptor inhibitor BAY 43-9006 in patients with advanced refractory solid tumors. J Clin Oncol.

[B41] Stippel DL, Kasper HU, Schleimer K, Tox U, Bangard C, Holscher AH, Beckurts KT (2005). Successful use of sirolimus in a patient with bulky ovarian metastasis of hepatocellular carcinoma after liver transplantation. Transplant Proc.

[B42] Ganten TM, Koschny R, Sykora J, Schulze-Bergkamen H, Buchler P, Haas TL, Schader MB, Untergasser A, Stremmel W, Walczak H (2006). Preclinical differentiation between apparently safe and potentially hepatotoxic applications of TRAIL either alone or in combination with chemotherapeutic drugs. Clin Cancer Res.

[B43] Avila MA, Berasain C, Sangro B, Prieto J (2006). New therapies for hepatocellular carcinoma. Oncogene.

[B44] Ganten TM, Haas TL, Sykora J, Stahl H, Sprick MR, Fas SC, Krueger A, Weigand MA, Grosse-Wilde A, Stremmel W, Krammer PH, Walczak H (2004). Enhanced caspase-8 recruitment to and activation at the DISC is critical for sensitisation of human hepatocellular carcinoma cells to TRAIL-induced apoptosis by chemotherapeutic drugs. Cell Death Differ.

[B45] Opferman JT, Iwasaki H, Ong CC, Suh H, Mizuno S, Akashi K, Korsmeyer SJ (2005). Obligate role of anti-apoptotic MCL-1 in the survival of hematopoietic stem cells. Science.

[B46] Fisher DE (1994). Apoptosis in cancer therapy: crossing the threshold. Cell.

[B47] Nerenstone SR, Ihde DC, Friedman MA (1988). Clinical trials in primary hepatocellular carcinoma: current status and future directions. Cancer Treat Rev.

[B48] Choi TK, Lee NW, Wong J (1984). Chemotherapy for advanced hepatocellular carcinoma. Adriamycin versus quadruple chemotherapy. Cancer.

[B49] Shipp MA, Ross KN, Tamayo P, Weng AP, Kutok JL, Aguiar RC, Gaasenbeek M, Angelo M, Reich M, Pinkus GS, Ray TS, Koval MA, Last KW, Norton A, Lister TA, Mesirov J, Neuberg DS, Lander ES, Aster JC, Golub TR (2002). Diffuse large B-cell lymphoma outcome prediction by gene-expression profiling and supervised machine learning. Nat Med.

[B50] Berrieman HK, Smith L, O'Kane SL, Campbell A, Lind MJ, Cawkwell L (2005). The expression of Bcl-2 family proteins differs between nonsmall cell lung carcinoma subtypes. Cancer.

[B51] Letai A (2005). Pharmacological manipulation of Bcl-2 family members to control cell death. J Clin Invest.

[B52] Zhou P, Qian L, Kozopas KM, Craig RW (1997). Mcl-1, a Bcl-2 family member, delays the death of hematopoietic cells under a variety of apoptosis-inducing conditions. Blood.

[B53] Coleman WB (2003). Mechanisms of human hepatocarcinogenesis. Curr Mol Med.

[B54] Philip PA, Mahoney MR, Allmer C, Thomas J, Pitot HC, Kim G, Donehower RC, Fitch T, Picus J, Erlichman C (2005). Phase II study of Erlotinib (OSI-774) in patients with advanced hepatocellular cancer. J Clin Oncol.

[B55] Fire A, Xu S, Montgomery MK, Kostas SA, Driver SE, Mello CC (1998). Potent and specific genetic interference by double-stranded RNA in Caenorhabditis elegans. Nature.

[B56] Romano PR, McCallus DE, Pachuk CJ (2006). RNA interference-mediated prevention and therapy for hepatocellular carcinoma. Oncogene.

[B57] Song E, Lee SK, Wang J, Ince N, Ouyang N, Min J, Chen J, Shankar P, Lieberman J (2003). RNA interference targeting Fas protects mice from fulminant hepatitis. Nat Med.

[B58] Wacheck V, Zangemeister-Wittke U (2006). Antisense molecules for targeted cancer therapy. Crit Rev Oncol Hematol.

[B59] Heere-Ress E, Thallinger C, Lucas T, Schlagbauer-Wadl H, Wacheck V, Monia BP, Wolff K, Pehamberger H, Jansen B (2002). Bcl-X(L) is a chemoresistance factor in human melanoma cells that can be inhibited by antisense therapy. Int J Cancer.

[B60] Han J, Goldstein LA, Gastman BR, Rabinowich H (2006). Interrelated roles for Mcl-1 and BIM in regulation of TRAIL-mediated mitochondrial apoptosis. J Biol Chem.

